# Supplementary material for: Optogenetic storage and release of protein and mRNA in live cells and animals
Source: Nat Commun. 2025 Jul 7;16:6230. doi: 10.1038/s41467-025-61322-y (PMC12234719; doi:10.1038/s41467-025-61322-y)
Supplement: Supplementary file 1 — Supplementary Information [file 41467_2025_61322_MOESM1_ESM.pdf]

Supplementary information

Optogenetic Storage and Release of Protein and mRNA in Live Cells and Animals

Chaeyeon Lee[1], Jeonghye Yu[1], Jongpil Shin[1], Jihwan Yu[1], Yungyeong Heo[1], Moosung Lee[2,3], Daseuli Yu[4*], YongKeun Park[2,3,5*] & Won Do Heo[1,6,7*]

[1]Department of Biological Sciences, Korea Advanced Institute of Science and Technology (KAIST), Daejeon 34141, Republic of Korea

[2]Department of Physics, Korea Advanced Institute of Science and Technology (KAIST), Daejeon, 34141, South Korea

[3]KAIST Institute for Health Science and Technology, KAIST, Daejeon, 34141, South Korea

[4]Life Science Research Institute, KAIST, Daejeon, Republic of Korea.

[5]Tomocube Inc., Daejeon, 34127, South Korea

[6]Department of Brain & Cognitive Sciences, KAIST, Daejeon 34141, Republic of Korea.

[7]KAIST Institute for the BioCentury (KIB), KAIST, Daejeon 34141, Republic of Korea.

*Correspondence: D.Y. (e-mail: sureeyu@kaist.ac.kr), Y.P. (e-mail: yk.park@kaist.ac.kr), W.D.H. (e-mail: wondo@kaist.ac.kr)

This file includes:

Supplementary Fig 1 to 13 and figures legends

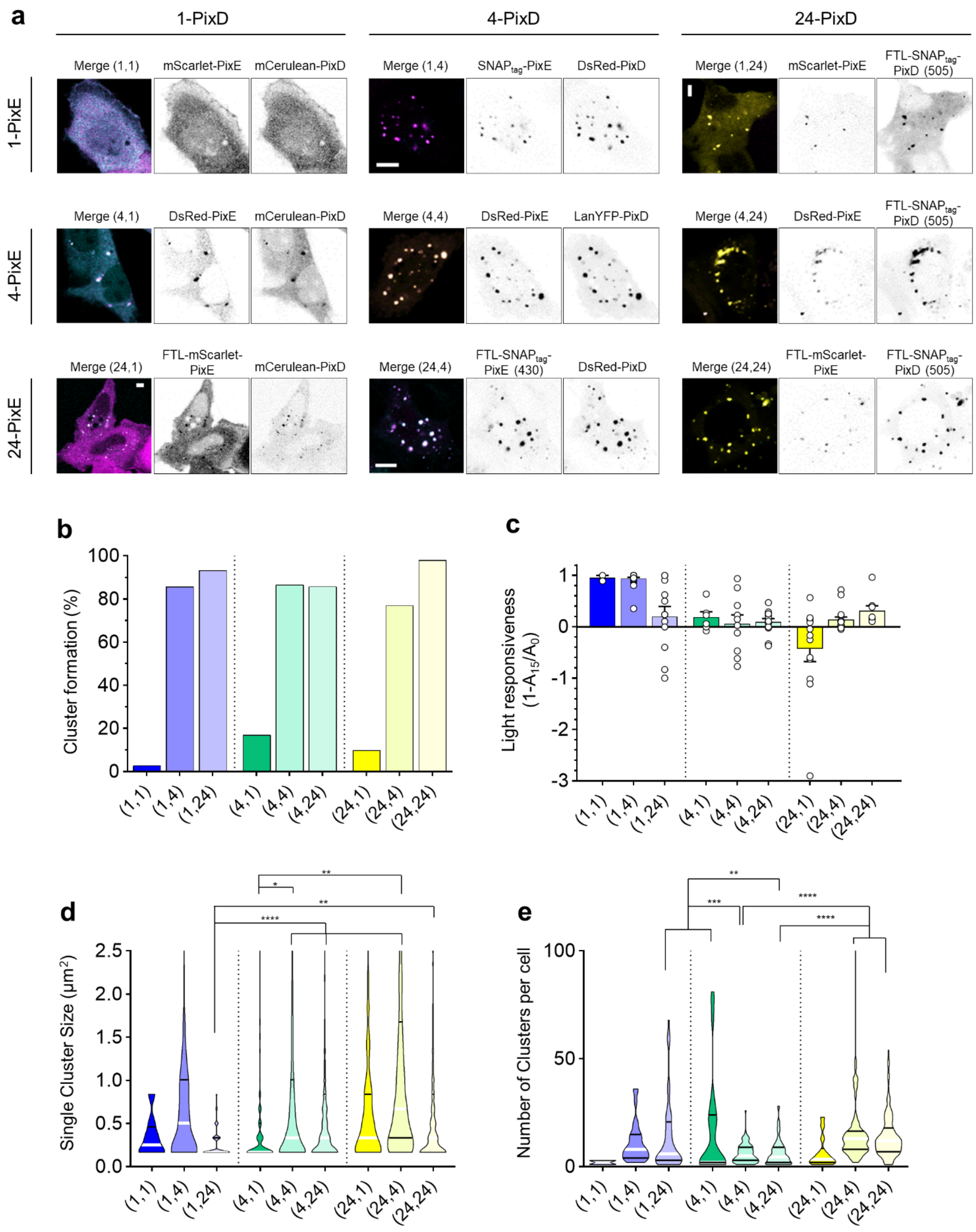

Supplementary Figure 1. Design of optogenetic condensate using multivalent modules

a. Fluorescence images of HeLa cells expressing nine combinations of container modules. Three container modules each for PixD (1-PixD, 4-PixD, and 24-PixD) and PixE (1-PixE, 4-PixE, and 24-PixE) were tested via expression in HeLa cells. Merge channel, shows inverted mono channel for PixE and PixD for each

combination. Scale bar, 10 μm.

b. Bar graph for cluster formation rate (%). Cluster formation rate was calculated as: (cluster formed - cells/cells expressing both PixD and PixE) x100(%). The number in parentheses indicate the number of oligomers formed by proteins conjugated to PixE and PixD, respectively. (eg). (1,1) for monomer-PixE with monomer-PixD. (n = 67, 80, 52, 152, 96, 99, 140, 78, 149 cells from the left)

c. Bar graph for light responsiveness. Light responsiveness was calculated by the area fold change of clusters after 15 min of light stimulation ($1-A_{15}/A_0$). (n= 2, 27, 12, 6, 10, 13, 13, 17, 8 cells)

d. Truncated violin plot for single cluster size (μm^2). White bold line, median; black line, quartiles. (n= 8, 61, 789, 428, 1064, 1377, 89, 1289, 2084 clusters from the left. **P=0.0032, *P=0.0362, **P=0.0062, ****P<0.0001 by one-way ANOVA, error bars, s.e.m.).

e. Truncated violin plot for cluster number per cell. White bold line, median; black line, quartiles. (n = 2, 27, 56, 26,82,90,14,69,146 cells from the left. **P= 0.0037, 0.0043, 0.0037, 0.0044, ****P<0.0001, by one-way ANOVA, error bars, s.e.m.)

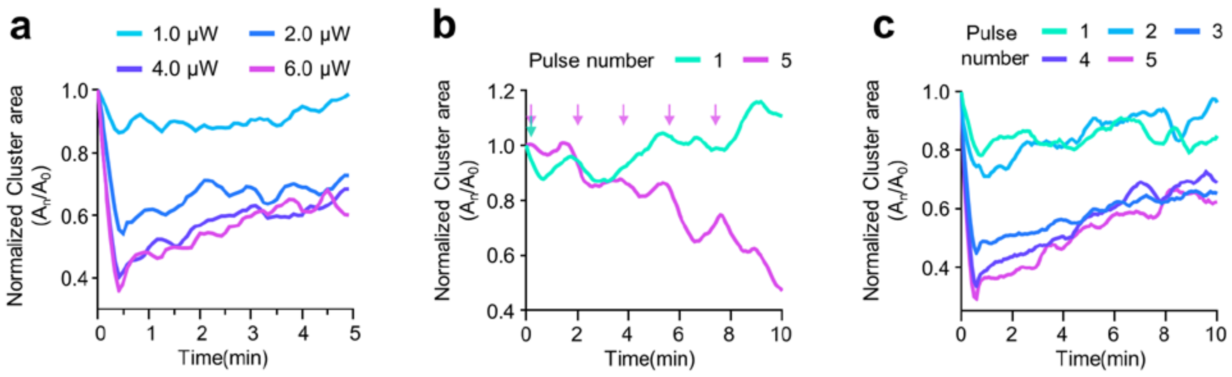

Supplementary Figure 2. Various Light Stimulation Conditions for RELISR

a. Time-lapse graph of cluster dissociation under different laser intensities. Light was applied as two pulses for each stimulation, with a 5-sec interval.

b. Time-lapse graph of cluster dissociation under pulsatile or sustained light stimulation. The laser intensity was fixed at 6.0 μW. Each pulse lasted 500 msec. For multiple pulse stimulation, 2-min intervals were applied between pulses. The acquisition interval was 5 sec and the acquisition duration was 10 min.

c. Time-lapse graph of cluster dissociation under different light-pulse numbers. The laser intensity was fixed at 6.0 μW. Each pulse lasted 500 msec. For multiple pulse stimulation, the following stimulation started immediately after the end of the prior pulse.

Each experiment was carried out in a single cell. To ensure that the conditions remained the same, the cell was pre-stimulated before the experiment and allowed to recover in the dark for 1 hr before being used in the next trial. Cluster area (A_n) represents the the sum of area fractions occupied by cluster at the time point of n min. Data are representative of three trials for each condition.

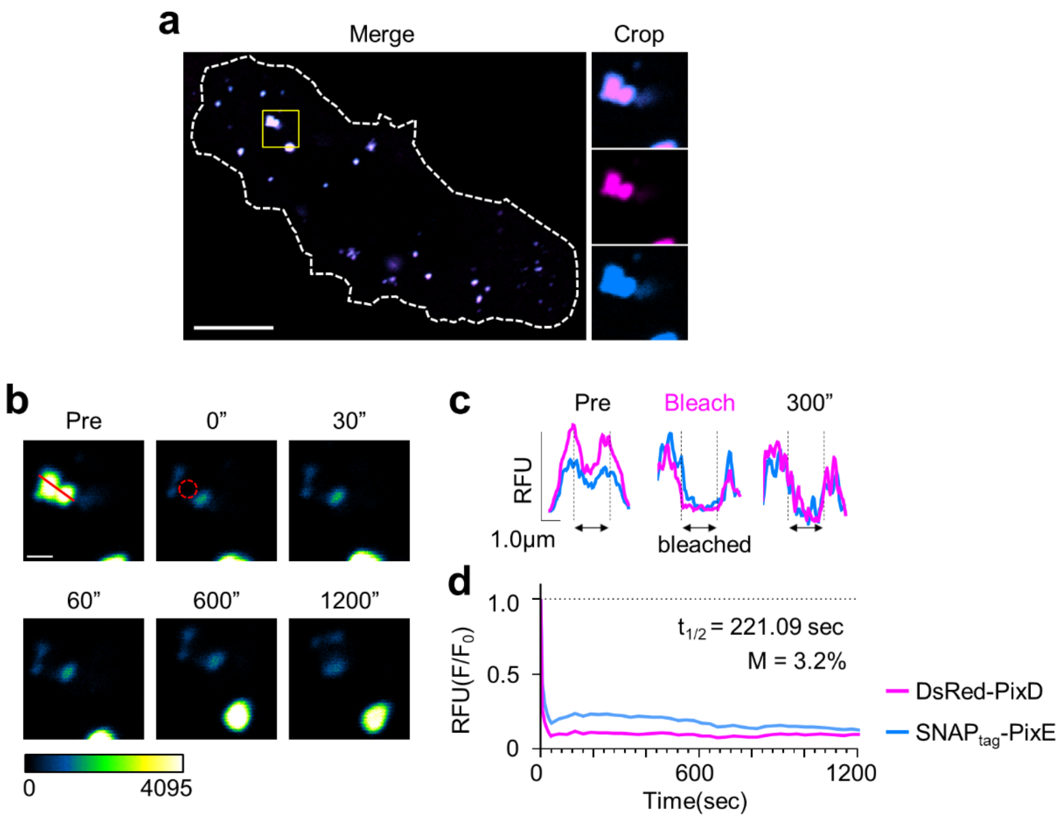

Supplementary Figure 3. RELISR Signal Shows Minimal Recovery in FRAP Experiments

a. Fluorescence images of HeLa cells expressing DsRed-PixD and $SNAP_{tag}$-PixE. Yellow box indicates the cropped area. White dotted line indicates the outline of the cell. (Crop) From top to bottom: Merge, DsRed, and $SNAP_{tag}$ (Alexa 647). Scale bar, 10 μm.

b. Fluorescence images of DsRed-PixD during FRAP experiments, pseudo-colored via look up table (LUT) (green-blue fire). LUTs range from 0 (blue) to 4095 (green). Red line indicates where intensity profiles were analyzed. Red dotted circle indicates the photobleached area within the cluster.

c. Intensity profiles for DsRed-PixD and $SNAP_{tag}$-PixE. "Pre" indicates the session preceding photobleaching. "Bleach" (magenta) was obtained immediately after photobleaching. "300 s" shows the intensity profile obtained 300 sec after photobleaching.

d. Time-lapse graph of fold changes in fluorescent intensity during FRAP experiments. The DsRed-PixD signal was used to calculate half-recovery time and mobile fraction. Half-recovery time ($t_{1/2}$) and the mobile fraction (M) was calculated by the equations in methods.

Each experiment was carried out in a single cell. Data are representative of three trials for each condition.

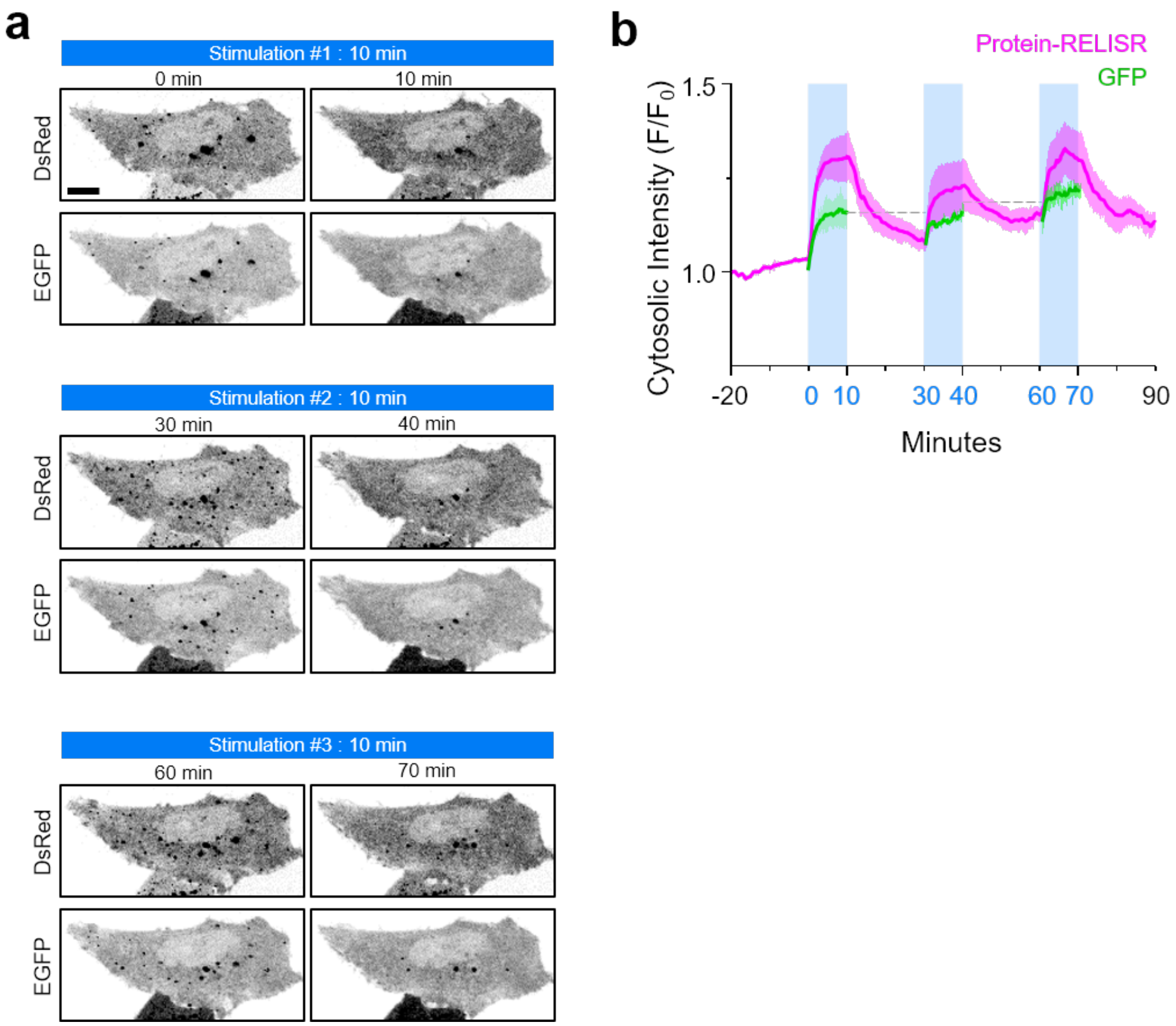

Supplementary Figure 4. Reversible Storage and Release of GFP by Protein-RELISR upon Repeated Light Stimulation

a. Fluorescence images of HeLa cells co-expressing GFP and Protein-RELISR during each light stimulation session. Blue light was applied for 10 minutes with 10-second intervals, and co-imaging of EGFP (488 nm) and DsRed (Protein-RELISR) was performed during illumination. Scale bar 10μm.

b. Time-lapse graph showing cytosolic fluorescence intensity for each channel: DsRed (magenta) and EGFP (green). EGFP intensity was acquired only during the light stimulation periods. Shaded regions represent the standard error of the mean (s.e.m) n = 3.

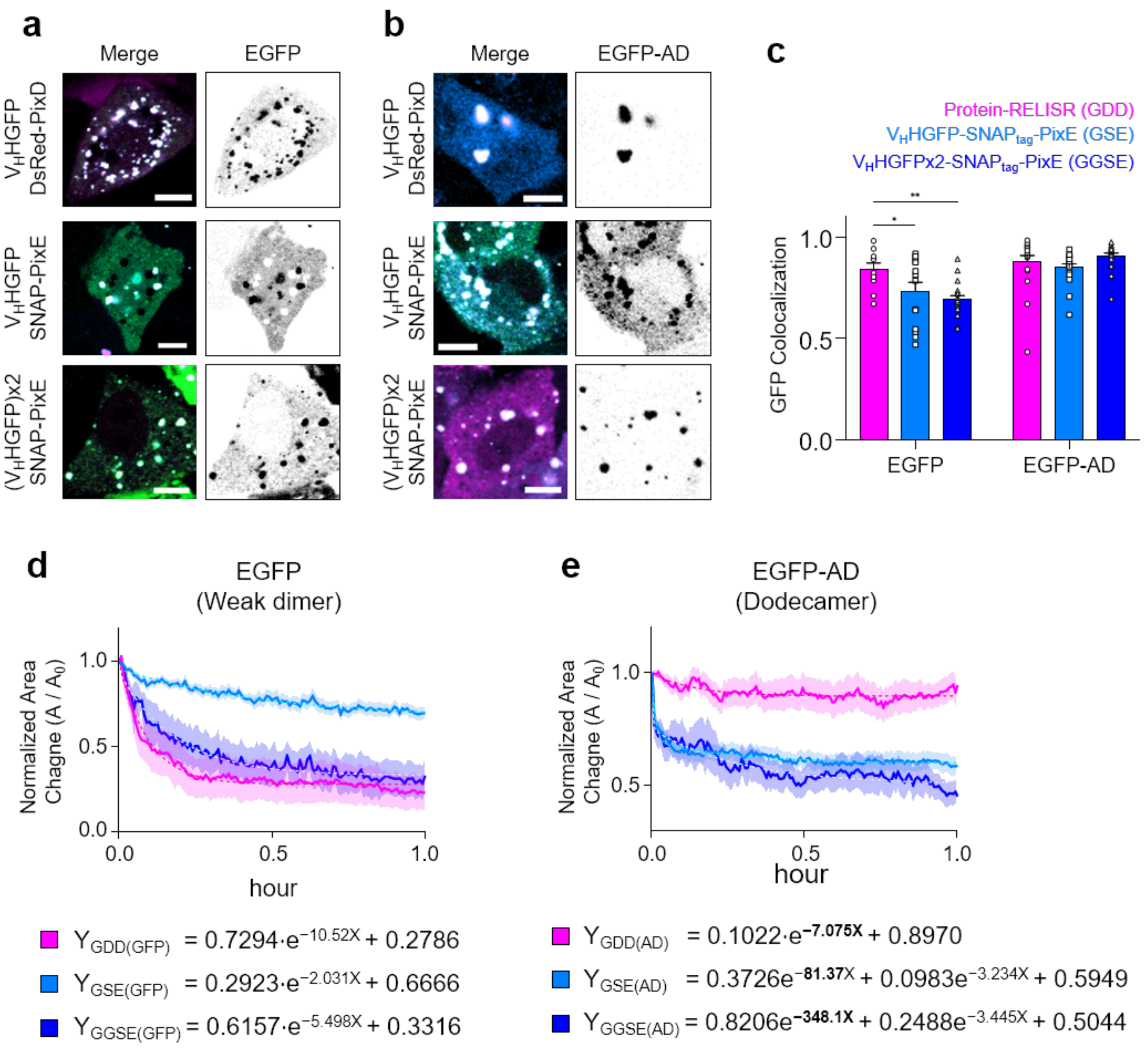

Supplementary Figure 5. Reversible Storage and Release of GFP by Protein-RELISR upon Repeated Light Stimulation

a. Fluorescence images of HeLa cells expressing monomeric EGFP with Protein-RELISR, VHHGFP-SNAP-PixE (GSE), or tandem nanobody construct VHHGFP-VHHGFP-SNAP-PixE (GGSE), Scale bar 10μm.

b. Fluorescence images of HeLa cells expressing multimeric EGFP-AD with the same three Protein-RELISR variants (Protein-RELISR, GSE, GGSE).

c. Colocalization analysis using Pearson correlation between EGFP or EGFP-AD and each RELISR construct channel: GDD (DsRed, magenta), GSE (SNAP-tag labeled with Alexa Fluor 647, sky blue), and GGSE (SNAP-tag, deep blue). Analysis was performed across entire cells. Statistical comparisons via two-way ANOVA: GDD vs. GSE (*P = 0.0375) and GDD vs. GGSE (P = 0.0013), both for GFP cargo. From the left,

sample sizes are n = 10, 14, 20, 20, 20, and 20. Error bars represent s.e.m.

d. Normalized time-lapse graph showing the light-induced dissociation of clusters targeting EGFP. Cells were illuminated with 488 nm light at 1.5 μW intensity for one hour with 30-second intervals. The Y-axis represents the normalized cluster area at each time point, calculated relative to the initial cluster area. One-phase decay model fitting was applied to each dataset using the equation: $Y = A \cdot e^{(-Kx)} + \text{Plateau}$, where A represents the initial amplitude, K is the decay rate constant, and Plateau indicates the residual cluster area. Shaded regions represent the standard error of the mean (s.e.m.), and dotted lines indicate fitted curves (red: GDD, sky blue: GSE, white: GGSE). Sample sizes: n = 9 (GDD), 12 (GSE), and 6 (GGSE).

e. Normalized time-lapse graph. Dissociation kinetics of clusters targeting EGFP-AD, plotted as normalized cluster area over time. Experimental conditions and model fitting follow (d). Dotted lines indicate fitted curves (sky blue: GSE, white: GGSE, red: GDD); shaded regions represent s.e.m. Sample sizes: n = 20 (GDD), 31 (GSE), and 15 (GGSE).

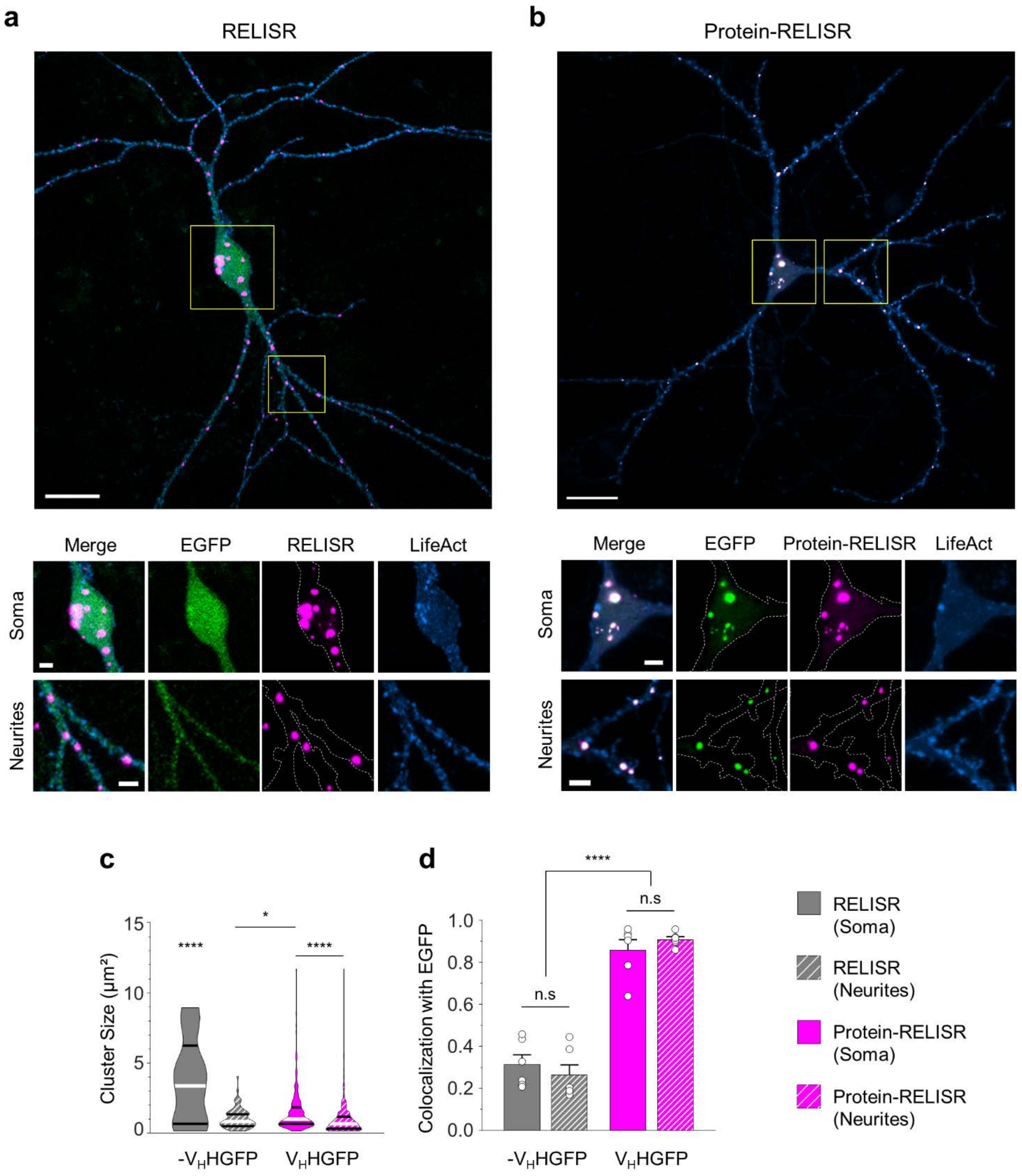

Supplementary Figure 6. RELISR and Protein-RELISR Form Clusters in Neurons

a, b. (Top of **a**) Fluorescence image of merged channels showing RELISR, EGFP, and LifeAct expressed in a whole neuron. (Top of **b**) Fluorescence image of merged channels showing Protein-RELISR, EGFP, and LifeAct expressed in a whole neuron. Yellow boxes indicate the cropped areas. Scale bar, 25 μm. (Bottom) Cropped images from the top panels. White dotted lines show the outline of neurons. Cropped image scale bar, 5 μm.

c. Truncated violin plot of cluster sizes of RELISR (annotated as "-V_HHGFP", gray) or Protein-RELISR (annotated as "V_HHGFP", magenta) distributed in soma and neurites. RELISR in soma formed significantly larger clusters than the other groups (****P<0.0001, *P=0.0280, ****P<0.0001, one-way ANOVA, error bars, s.e.m., n=9, 79, 245, 436 clusters for each group). White bold line, median; black line, quartiles.

d. Colocalization analysis of DsRed signals with EGFP signals (n=6 per cell, ****P<0.0001, one-way ANOVA, error bars, s.e.m.).

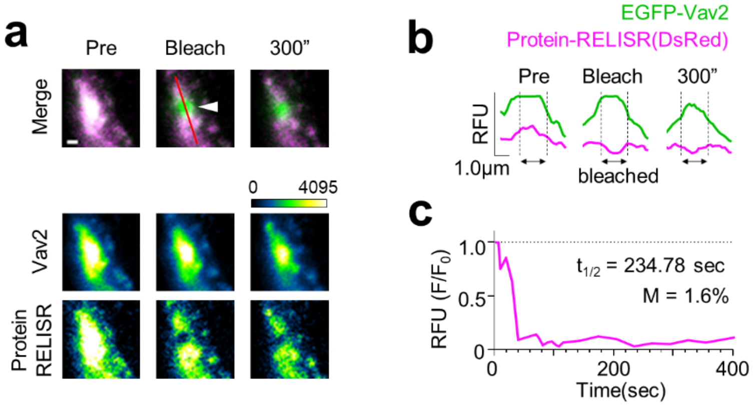

Supplementary Figure 7. GFP-Vav2 Targeting Protein-RELISR Showed Low Recovery Rates on FRAP

a. Fluorescence images of Protein-RELISR and EGFP-Vav2 obtained during FRAP experiments. Merged channel images are presented in color (magenta, V_HHGFP-DsRed-PixD; green, EGFP). Single channels of Vav2 and Protein-RELISR are pseudo-colored via look up table (LUT) (green-blue fire). LUTs range from 0 (blue) to 4095 (green). Red line indicates where the intensity profiles were analyzed. White arrow indicates the bleached area.

b. Intensity profiles for EGFP and DsRed. "Pre" indicates the session preceding photobleaching. "Bleach" (magenta) shows data obtained right after photobleaching. "300 s" shows the intensity profile 300 sec after photobleaching.

c. Time-lapse graph of fold changes in fluorescent intensity. The V_HHGFP-DsRed-PixD signal was used to calculate half-recovery time and mobile fraction. Half-recovery time (t1/2) and the mobile fraction (M) was calculated by the equations in methods.

Each experiment was carried out in a single cell. Data are representative of three trials for each condition.

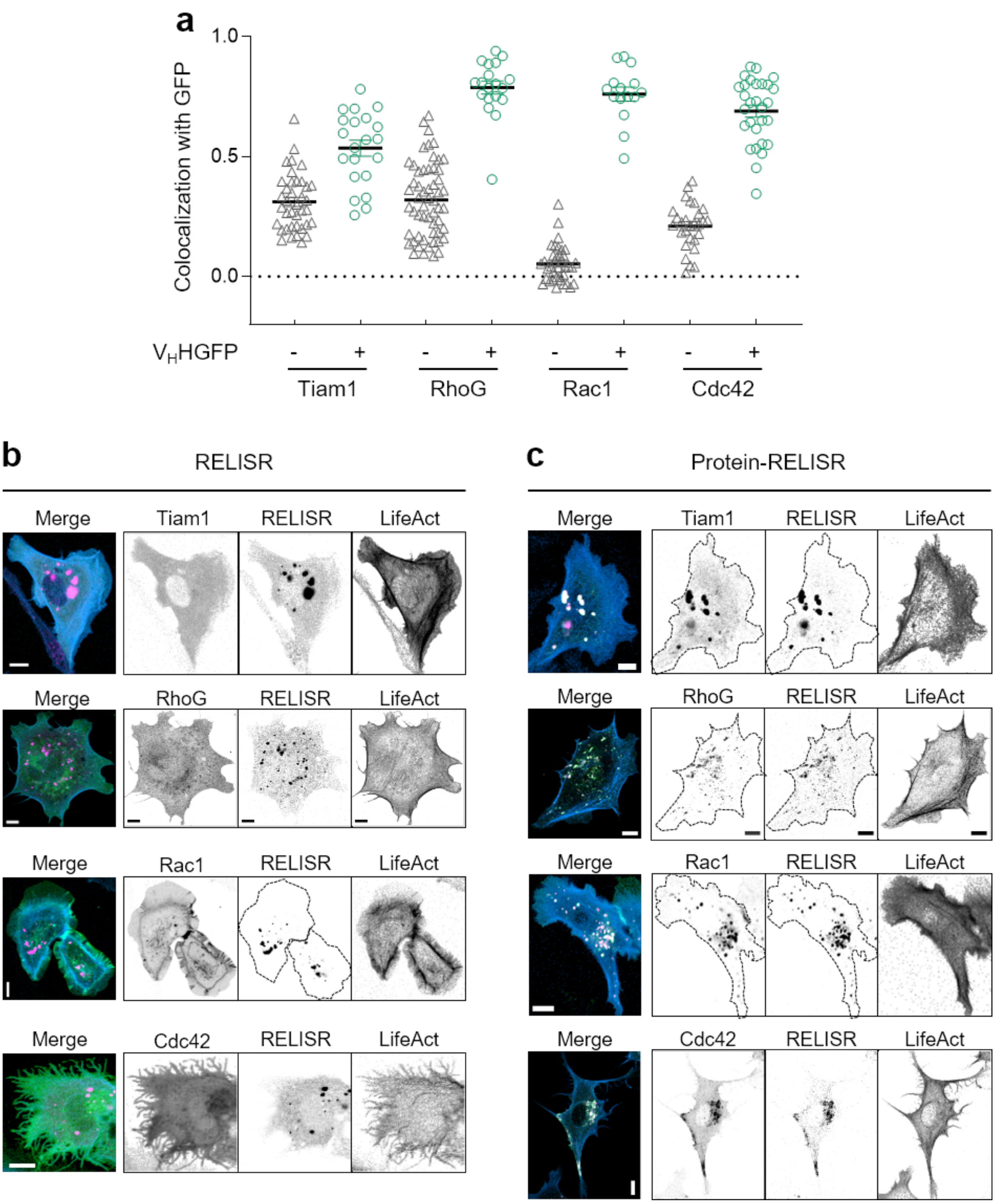

Supplementary Figure 8. Protein-RELISR can trap Various GFP-Conjugated Proteins

a. Colocalization analysis of RELISR ("-V_HHGFP") and Protein-RELISR ("+V_HHGFP") with EGFP-conjugated proteins. Gray triangles indicate RELISR-expressing cells, and green circles indicate Protein-RELISR-expressing cells. Each symbol represents a single cell. The black bold line indicates the mean of the group. (Tiam1: n=38, 21, ****P<0.0001, t-test), (RhoG: n=56, 19, ****P<0.0001, t-test), (Rac1: n=37, 15, ****P<0.0001, t-test), (Cdc42: n=28, 20, ****P<0.0001, t-test).

b, c. Representative images of each group shown in panel **a**. Black dotted lines indicate outlines of cells.

EGFP-conjugated proteins (green), RELISR or Protein-RELISR (magenta), and LifeAct (blue) are shown in merged channel images. Inverted monochrome images are presented for each construct.

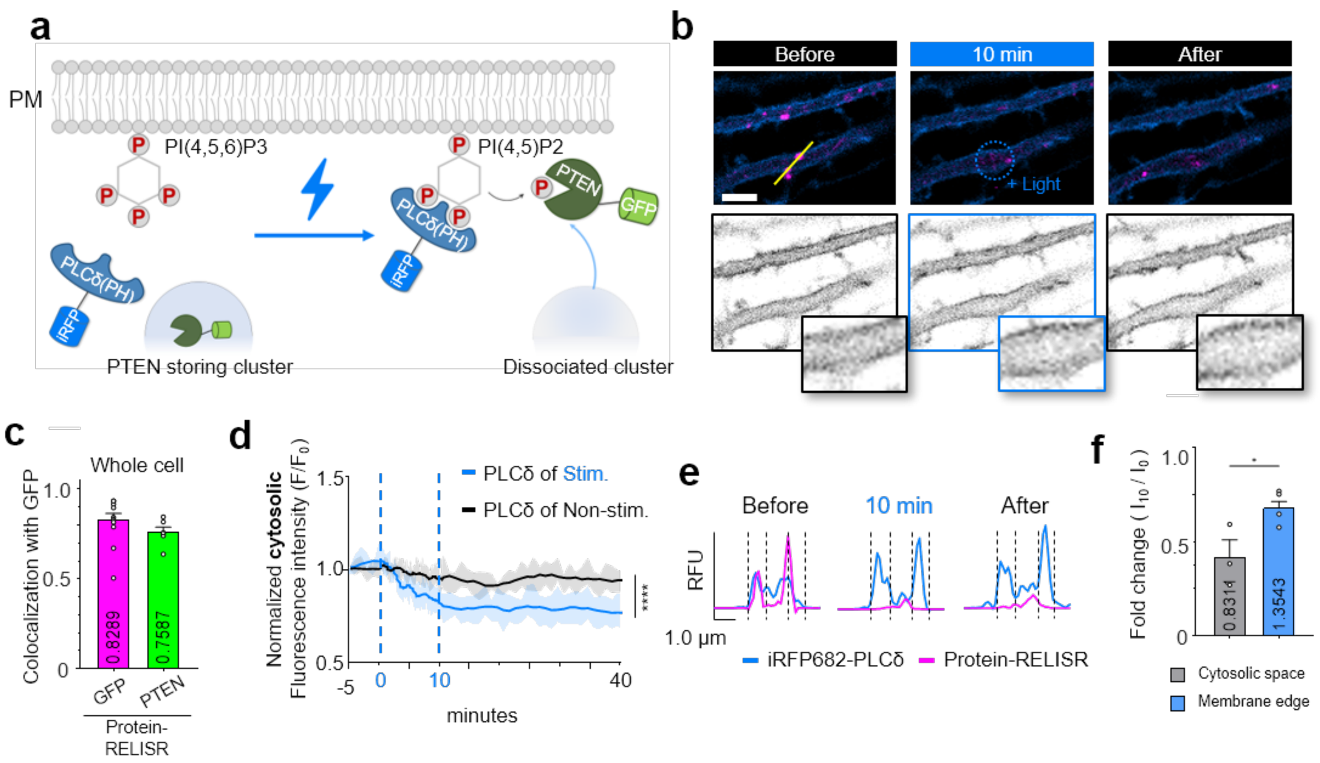

Supplementary Figure 9. Local release of PTEN from Protein-RELISR alters subcellular distribution of PLCδ PH domain in neurons.

a. Schematic representation of the PLCδ PH domain localization following local release of PTEN from Protein-RELISR. In the dark, PTEN is stored in the cluster, and PLCδ remains in the cytosol. Upon light-induced release, PTEN converts PIP_3 to PIP_2, promoting membrane binding of PLCδ PH domain.

b. Time-lapse images of neurites expressing Protein-RELISR, iRFP682–PLCδ PH domain, and PTEN–GFP (not shown). Images were acquired before light stimulation ("Before"), 10 minutes after stimulation ("10 min"), and following 30 minutes of recovery ("After"). Top panels show merged fluorescence channels; bottom panels display cropped monochrome images of the iRFP signal from the PLCδ PH domain. The yellow line indicates the region analyzed for the intensity profile in panel e, and the blue dashed circle marks the light stimulation ROI.

c. Colocalization analysis using Pearson correlation between GFP or PTEN–GFP and Protein-RELISR in neurons (n = 12 for GFP- magenta, n = 6 for PTEN–GFP-green; unpaired t-test, P = 0.2195).

d. Time-lapse graph of normalized iRFP682–PLCδ PH domain intensity (F/F_0). Fluorescence intensities were normalized to the initial value at time 0. The blue line represents the stimulated region, and the gray line

indicates a non-stimulated region. Shaded areas denote s.e.m. The light stimulation period (0–10 min) is indicated by a vertical blue dashed line. (n = 5, ****P < 0.0001)

e. Intensity profiles corresponding to panel b, captured before light stimulation ("Before"), 10 minutes after stimulation ("10 min"), and after 30 minutes of recovery ("After"). The blue line represents PLCδ (iRFP682) signal intensity, and the magenta line corresponds to Protein-RELISR (DsRed). The black dashed line indicates the distal boundary of the iRFP signal in the "Before" image. RFU denotes relative fluorescence units; values are not normalized. Scale bar, 1.0 μm.

f. Quantification of PLCδ intensity fold change (Light/Dark) after 10 minutes of light stimulation. Gray bars represent the cytosolic region between membrane edges; blue bars indicate membrane edges. Data are shown as fold change relative to the dark state (n = 3 for cytosol, n = 6 for membrane; unpaired t-test, *P = 0.0205). Error bars represent s.e.m.

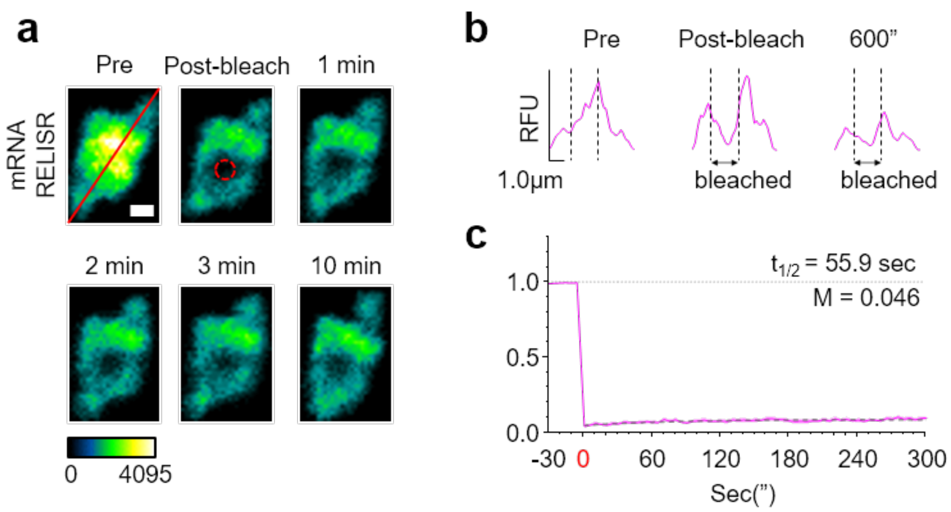

Supplementary Figure 10. FRAP Analysis of mRNA-RELISR Reveals Limited Internal Mobility of Clusters

a. Fluorescent images of FRAP experiments of mRNA-RELISR. mRNA-RELISR (DsRed) are pseudo-colored (green-blue fire), respectively. LUTs range from blue (0) to green (4095). The red line indicates where intensity profiles for panel **b** were analyzed. Scale bar, 1 μm.

b. Intensity profile of the bleached cluster. mRNA-RELISR (magenta). "Pre" indicates the session preceding photobleaching. "Bleach" (magenta) shows right after photobleaching. “600” corresponds to 10 minutes after photobleaching.

c. Time-lapse graph of FRAP experiments. DsRed (mRNA-RELISR) fluorescence intensities were normalized to the pre-bleach baseline. The graph includes 30 seconds prior to bleaching (“0” indicates the bleaching time point). Curve fitting and calculation of half-recovery time and mobile fraction were performed as described in the FRAP methods.

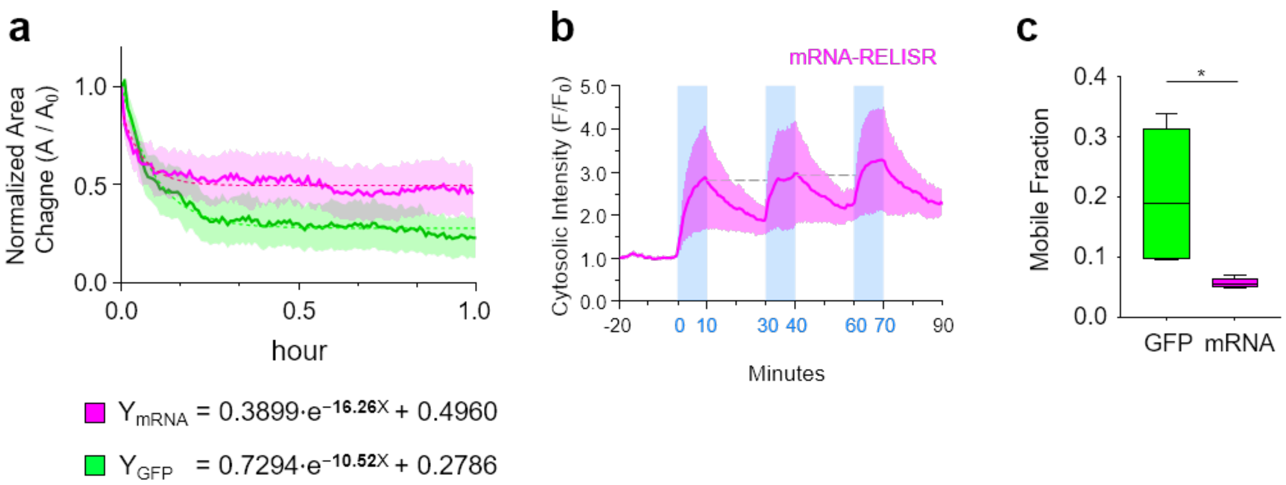

Supplementary Figure 11. Comparative dynamic analysis of Protein-RELISR and mRNA-RELISR.

a. Time-lapse graph of normalized area fraction of during light-stimulation of Protein-RELISR and mRNA-RELISR. Cells were illuminated with 488 nm light at 1.5 μW intensity for one hour with 30-second intervals. The Y-axis represents the normalized cluster area at each time point, calculated relative to the initial cluster area. One-phase decay model fitting was applied to each dataset using the equation: $Y = A \cdot e^{-Kx} + \text{Plateau}$, where A represents the initial amplitude, K is the decay rate constant, and Plateau indicates the residual cluster area. Shaded regions represent the standard error of the mean (s.e.m.), and dotted lines indicate fitted curves (green : Protein-RELISR, red: mRNA-RELISR). Sample sizes: n = 9 (Protein-RELISR), 9 (mRNA-RELISR).

b. Time-lapse graph showing cytosolic fluorescence intensity for mRNA-RELISR(DsRed, magenta). Shaded blue boxes indicate repeated light sessions. Shaded pink regions represent the standard error of the mean (s.e.m) n = 3.

c. Box-and-Whisker plot of mobile fractions. Green: Protein-RELISR; magenta : mRNA-RELISR. (Unpaired t-test, *P= 0.0186, each n= 5).

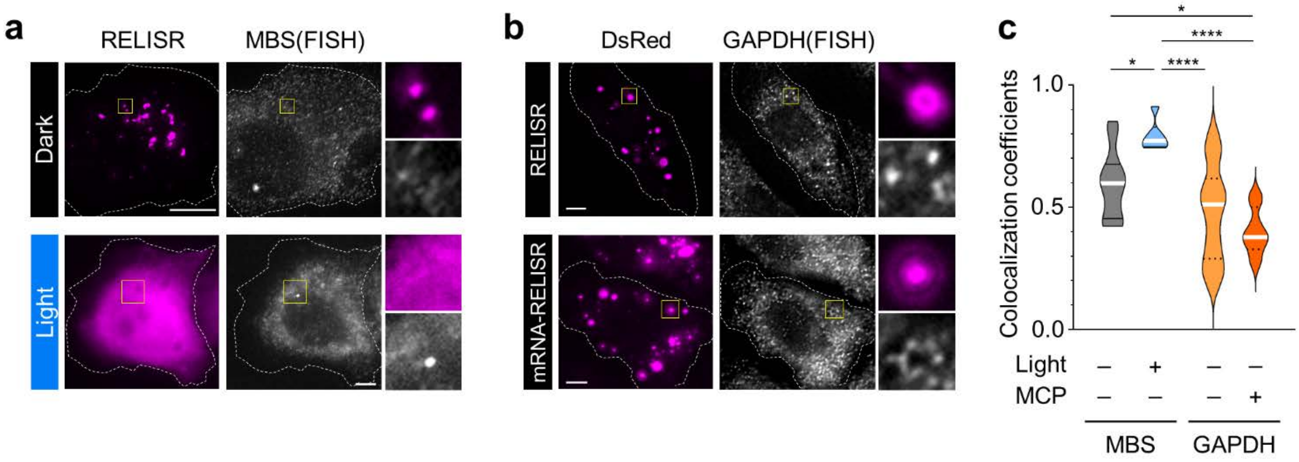

Supplementary Figure 12. MBS-Specific Trapping of mRNA-RELISR

a. Fluorescence images showing RELISR and MBS FISH signals in the dark and light-stimulated conditions. RELISR (magenta) was imaged through the G2A channel. MBS FISH signal was visualized using a Quasar 670-labeled FISH probe. Yellow boxes indicate the cropped area. White dotted lines indicate the outlines of cells. Scale bar, 10 μm.

b. Fluorescence images showing RELISR or mRNA-RELISR with GAPDH FISH signals in the dark state. DsRed signals (magenta) were imaged through the G2A channel. GAPDH mRNA signal was visualized using a Quasar 670-labeled FISH probe. Yellow boxes indicate the cropped area. White dotted lines indicate the outlines of cells. Scale bar, 10 μm.

c. Violin plot for colocalization analysis of results shown in panels **a** and **b**. For the two images on the left (colocalization with MBS FISH in panel **a**), gray represents RELISR in the dark and blue represents RELISR with light stimulation. For the two images on the right (colocalization with GAPDH FISH in panel **b**), pale orange represents RELISR in the dark and orange represents mRNA-RELISR in the dark (*P=0.0465, *P=0.0291, ****P<0.0001, ****P<0.0001, n=12, 6, 18, 8, one-way ANOVA).

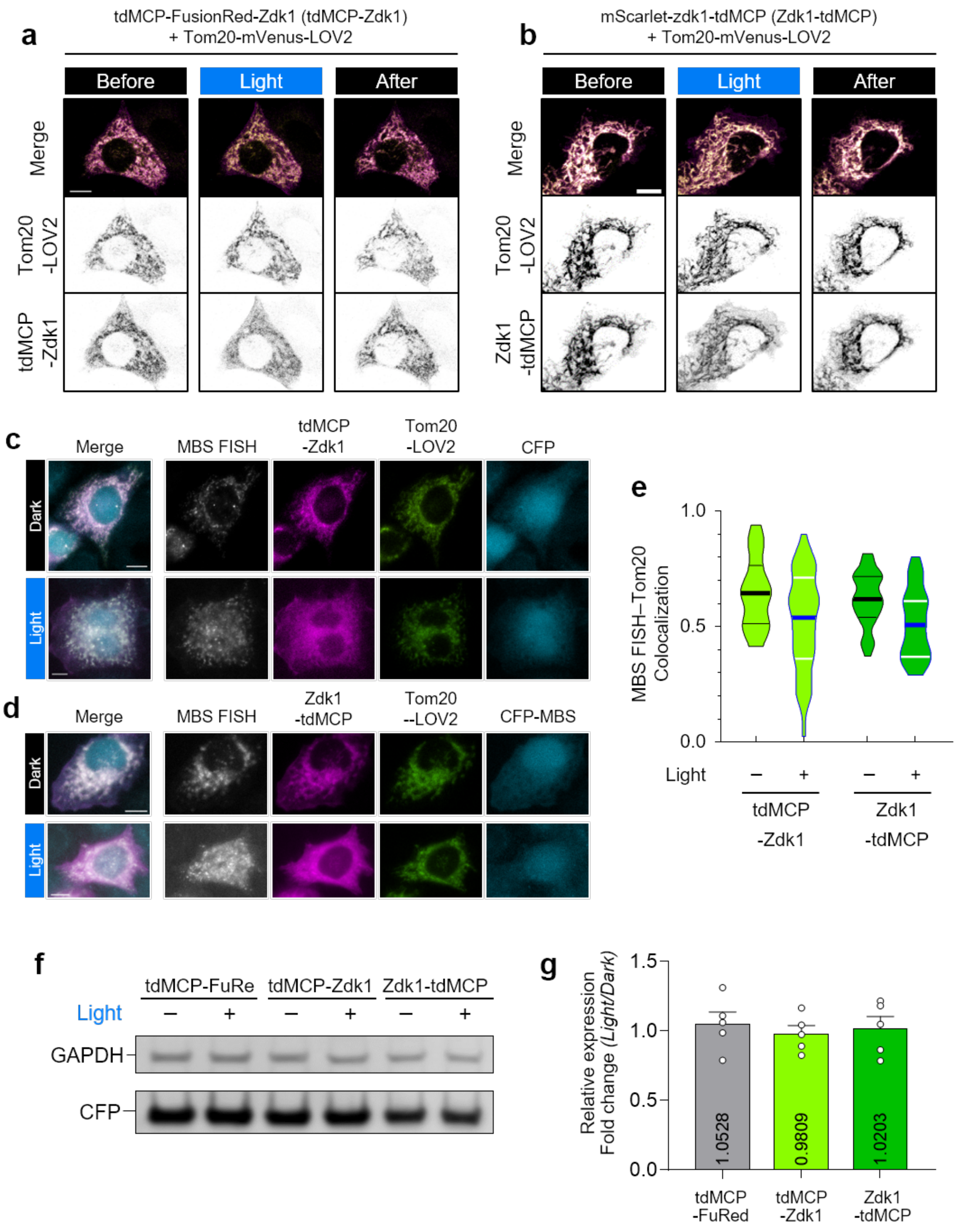

Supplementary Figure 13. LOVTRAP Is Insufficient for mRNA Retention and Translational Repression

a. Time-lapse images of HeLa cells expressing tdMCP–FusionRed–Zdk1 (tdMCP-Zdk1) and Tom20–mVenus–LOV2 (Tom20-LOV2). Images were acquired before light stimulation (“Before”), 30 minutes after stimulation (“Light”), and after a 1-hour recovery period (“After”). Top panels show merged fluorescence

channels; bottom panels display monochrome images of mVenus (Tom20-LOV2) and DsRed (tdMCP-Zdk1). Scale bar, 10 μm.

b. Time-lapse images of HeLa cells expressing mScarlet–Zdk1–tdMCP (Zdk1-tdMCP) and Tom20–mVenus–LOV2. Images were acquired as described in panel a. Top panels show merged fluorescence channels; bottom panels display monochrome images of mVenus (Tom20-LOV2) and DsRed (Zdk1-tdMCP). Scale bar, 10 μm.

c. Fluorescence images of tdMCP-Zdk1 and Tom20-LOV2 with MBS FISH signals under dark and light-stimulated conditions. MBS-tagged mRNAs were detected using Quasar 670-labeled FISH probes (2-sec exposure). tdMCP-Zdk1 (magenta) was imaged through the G2A channel (60–100 msec exposure), Tom20-LOV2 through the YFP channel (100 msec), and CFP via the CFP channel (100 msec). Scale bar, 10 μm.

d. Fluorescence images of Zdk1-tdMCP and Tom20-LOV2 with MBS FISH signals under the same imaging conditions as described in panel c.

e. Colocalization analysis (truncated violin plot) showing Pearson's correlation between MBS FISH signals and Tom20-LOV2 under dark and light conditions. One-way ANOVA showed no significant differences: tdMCP-Zdk1 (dark vs. light), $P = 0.0767$; Zdk1-tdMCP (dark vs. light), $P = 0.0960$; tdMCP-Zdk1 (dark) vs. Zdk1-tdMCP (dark), $P = 0.9576$. Sample sizes (left to right): $n = 15, 39, 35, 23$.

f. Immunoblot analysis of CFP protein expression under dark and light conditions. GAPDH (37 kDa) and CFP (27 kDa) were detected. "tdMCP-FuRed" indicates cells expressing CFP–MBSx24 and tdMCP–FusionRed. "tdMCP-Zdk1" and "Zdk1-tdMCP" indicate cells co-expressing CFP–MBSx24 with the respective Zdk1–tdMCP constructs and Tom20-LOV2. All samples were incubated under either dark or light-stimulated conditions for 24 hours.

g. Quantification of immunoblot results. CFP band intensities were normalized to GAPDH("Relative expression"), and fold changes (Light / Dark) were plotted as bar graphs. Gray: tdMCP-FuRed; light green: tdMCP-Zdk1; green: Zdk1-tdMCP. No significant differences were observed between groups (one-way ANOVA; $n = 5$ per group; $P = 0.7919, 0.9527, 0.9314$). Error bars represent s.e.m., and mean values are shown within each bar.

Full blot for Supplementary Figure 13g

*Red boxes indicate the regions cropped for each figure panel.

Full blot images corresponding to Fig. 5j and Supplementary Fig. 13g are provided in this document.

Loading Control (GAPDH, 37kDa) were included in every blot. (Invitrogen, MA5-15738, 1:1000)

Primary GFP antibody (Santa Cruz, SC-9996, 1:1000) were used to target CFP (27kDa).

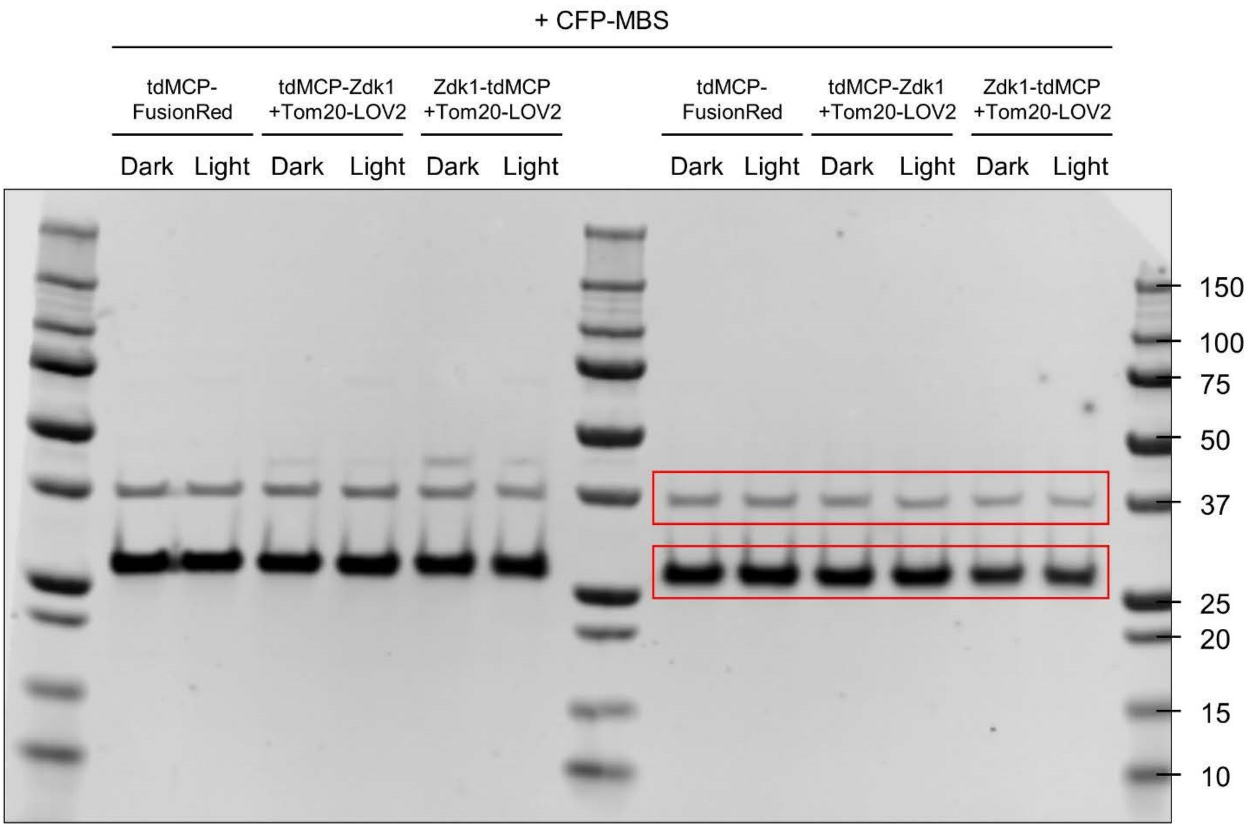

*tdMCP-Zdk1 : tdMCP-FusionRed-Zdk1;

*Zdk1-tdMCP : mScarlet-Zdk1-tdMCP;

*Tom20-LOV2 : Tom20-mVenus-LOV2

Samples on the left and right were prepared on different days but loaded together on the same gel.

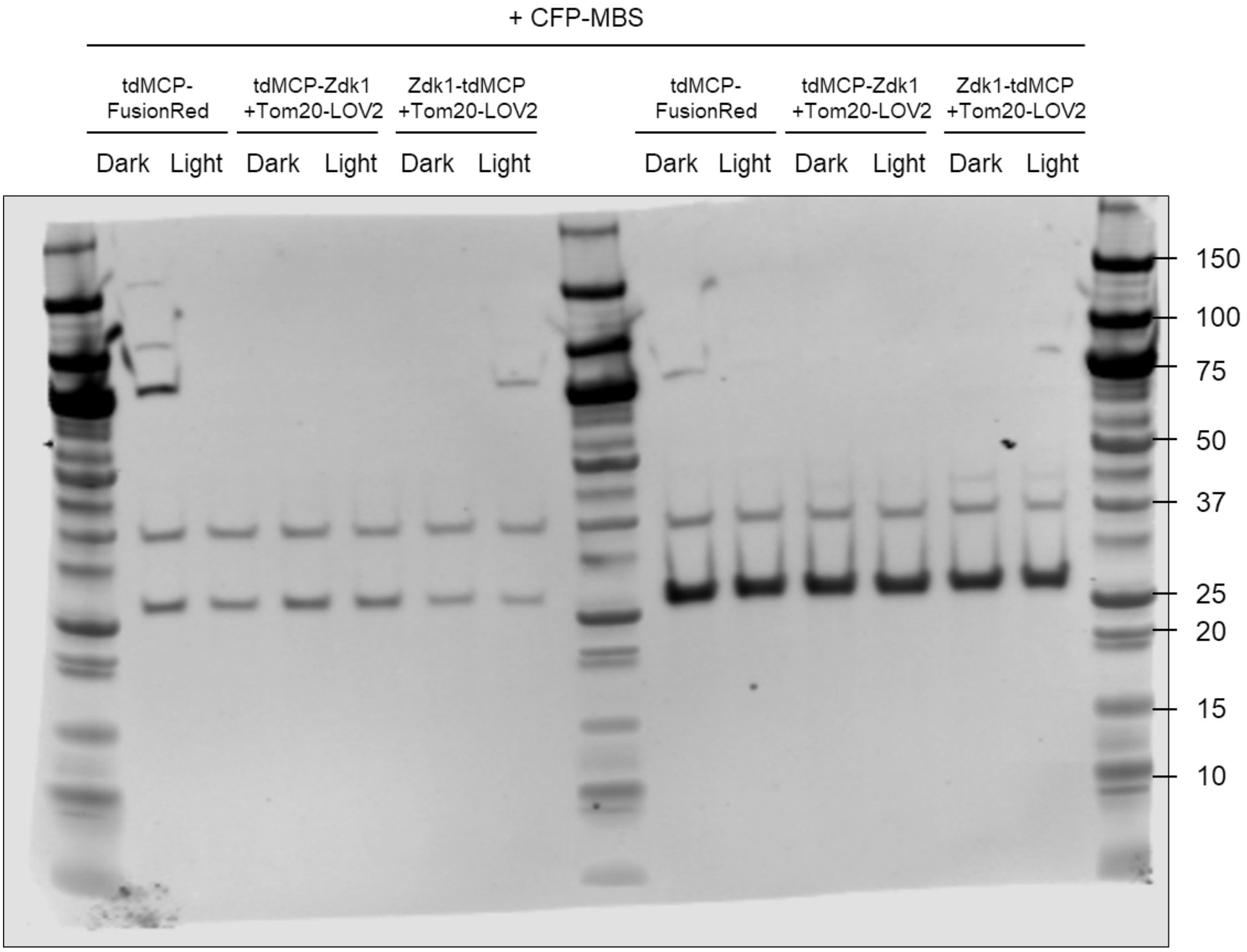

*tdMCP-Zdk1 : tdMCP-FusionRed-Zdk1;

*Zdk1-tdMCP : mScarlet-Zdk1-tdMCP;

*Tom20-LOV2 : Tom20-mVenus-LOV2

Samples on the left and right were prepared on different days but loaded together on the same gel.

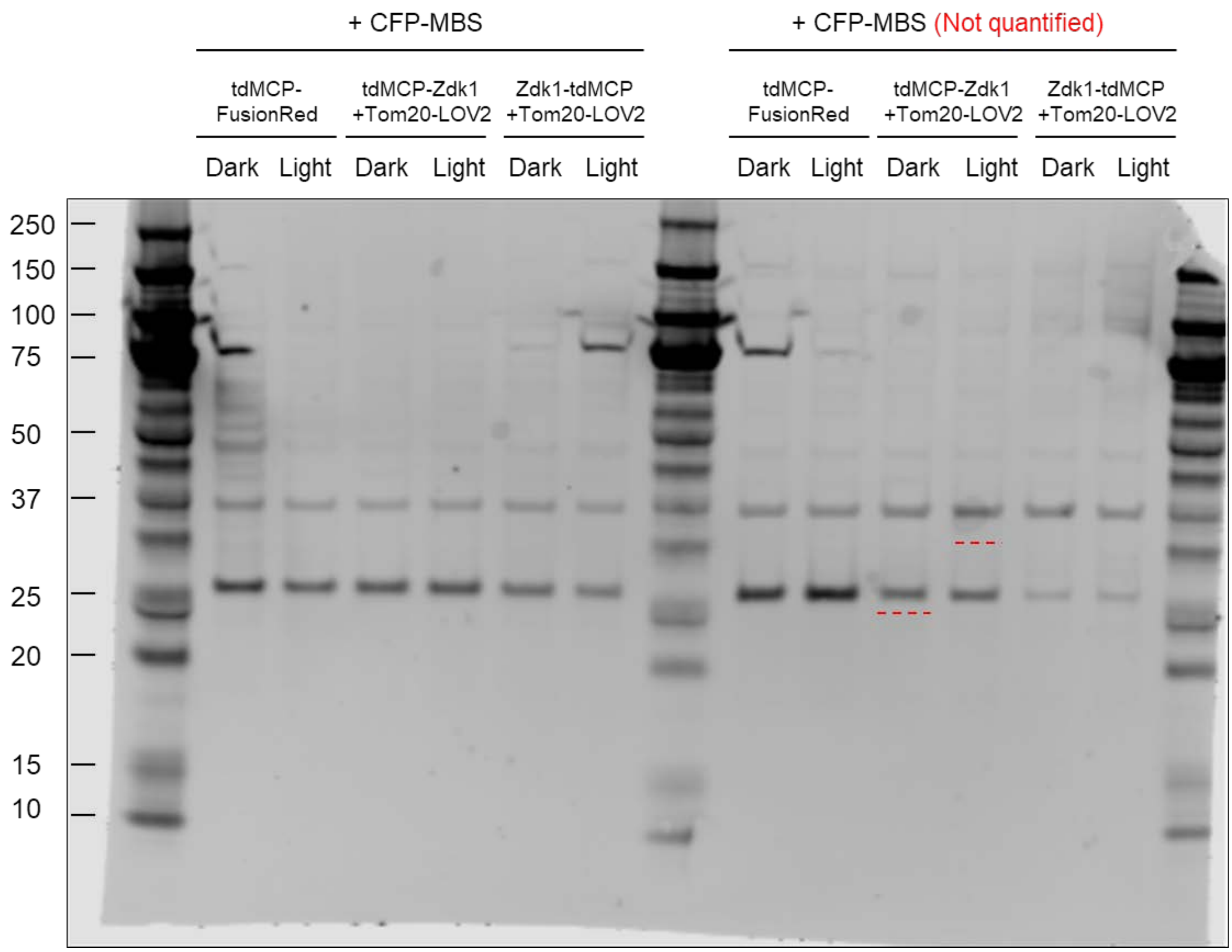

*tdMCP-Zdk1 : tdMCP-FusionRed-Zdk1;

*Zdk1-tdMCP : mScarlet-Zdk1-tdMCP;

*Tom20-LOV2 : Tom20-mVenus-LOV2

Samples on the left and right were prepared on different days but loaded together on the same gel.

Quantification of the right-side bands was omitted due to splotchy signal at the band region (red dashed line).
