# Supplementary material for: Optogenetic storage and release of protein and mRNA in live cells and animals
Source: Nat Commun. 2025 Jul 7;16:6230. doi: 10.1038/s41467-025-61322-y (PMC12234719; doi:10.1038/s41467-025-61322-y)
Supplement: Supplementary file 2 — Description of Additional Supplementary Files [file 41467_2025_61322_MOESM2_ESM.pdf]

## Description of Additional Supplementary Files

File Name: Supplementary Movie 1
Description: Merged-channel time-lapse of a HeLa cell expressing RELISR—DsRed-PixD and SNAPtag-PixE (corresponding to Fig. 1c). The cell was imaged and illuminated (488 nm) using a confocal microscope at 15-second intervals for 15 minutes. After illumination, imaging continued every 15 seconds for one hour. Scale bar, 10 µm.

File Name: Supplementary Movie 2
Description: Local light stimulation of HeLa cells expressing RELISR (inverted monochrome; corresponding to Fig. 1f). "S1:2" indicates the stimulated region. The movie shows the full 4-minute local stimulation session. Scale bar, 10 µm.

File Name: Supplementary Movie 3
Description: Local light stimulation of a hippocampal neuron expressing Protein-RELISR (VHHGFP–DsRed–PixD and SNAPtag–PixE) along with EGFP (corresponding to Fig. 3d–l). The movie shows merged channels, with EGFP and DsRed ("Protein-RELISR") displayed in inverted monochrome. Blue circles indicate the locally stimulated area. Scale bar, 10 µm.

File Name: Supplementary Movie 4
Description: Light-induced morphological changes in NIH3T3 fibroblasts co-expressing Protein-RELISR (magenta), EGFP–Vav2 (green), and iRFP–LifeAct (blue; corresponding to Fig. 4c). EGFP was imaged only during the 30-minute light stimulation period. Images were acquired every 15 seconds using a confocal microscope. Scale bar, 10 µm.

File Name: Supplementary Movie 5
Description: Light-responsive behavior of mRNA-RELISR. Whole-cell illumination of a HeLa cell expressing mRNA-RELISR and CFP–MBS (corresponding to Fig. 5f). The cell was imaged for one hour prior to light stimulation, followed by two hours of illumination and two additional hours of imaging. Images were acquired every 15 seconds using a confocal microscope. Scale bar, 10 µm.
