# Supplementary figures and images for: Optogenetic storage and release of protein and mRNA in live cells and animals
Source: Nat Commun. 2025 Jul 7;16:6230. doi: 10.1038/s41467-025-61322-y (PMC12234719; doi:10.1038/s41467-025-61322-y)

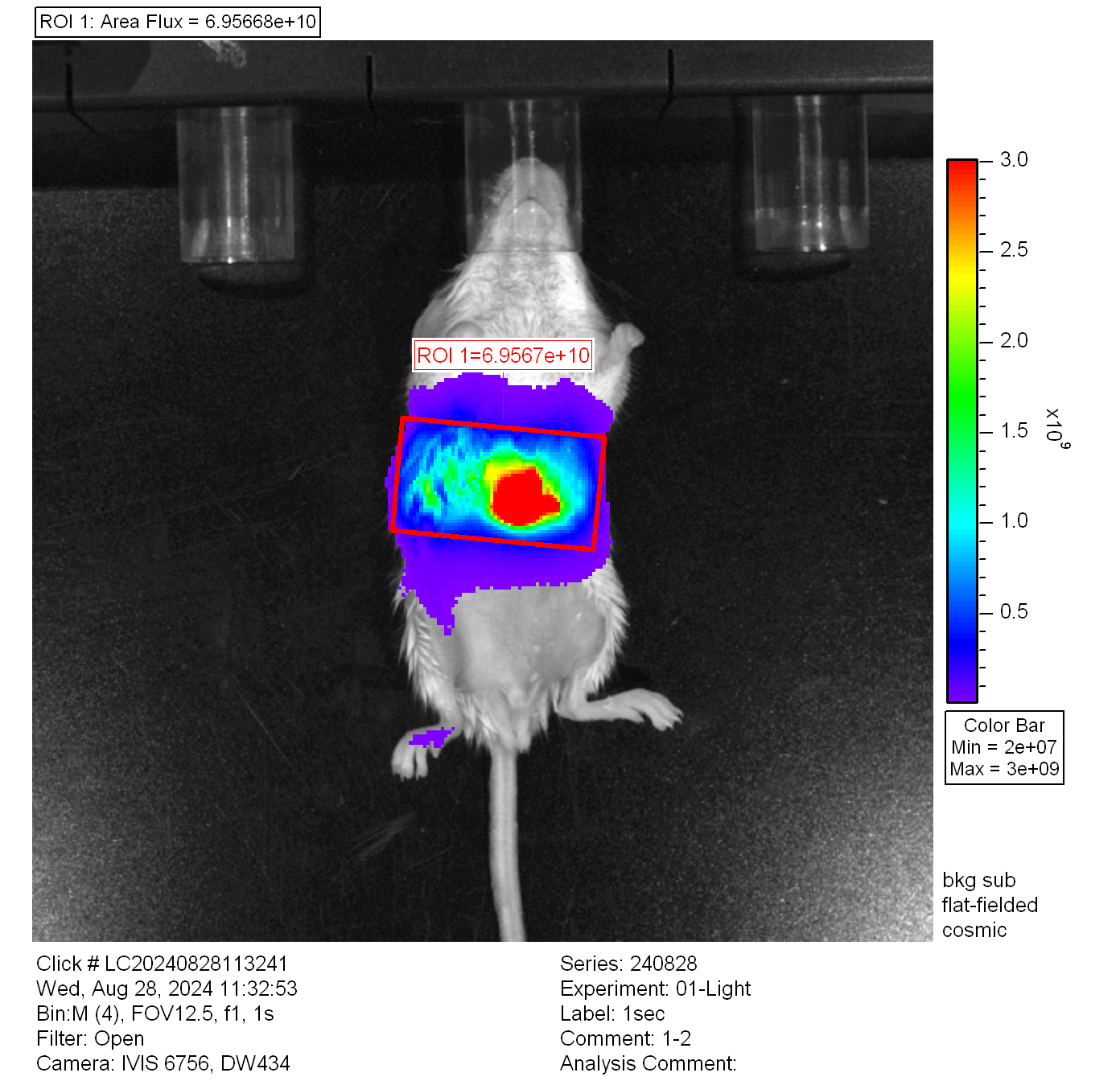

Supplement: Supplementary file 10 — Source Data [file 41467_2025_61322_MOESM10_ESM.zip › Source Data - Figure 6b/01_Positive control(Light)_composite.PNG]

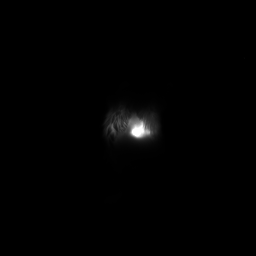

Supplement: Supplementary file 10 — Source Data [file 41467_2025_61322_MOESM10_ESM.zip › Source Data - Figure 6b/01_Positive control(Light)_Luminescent.TIF]

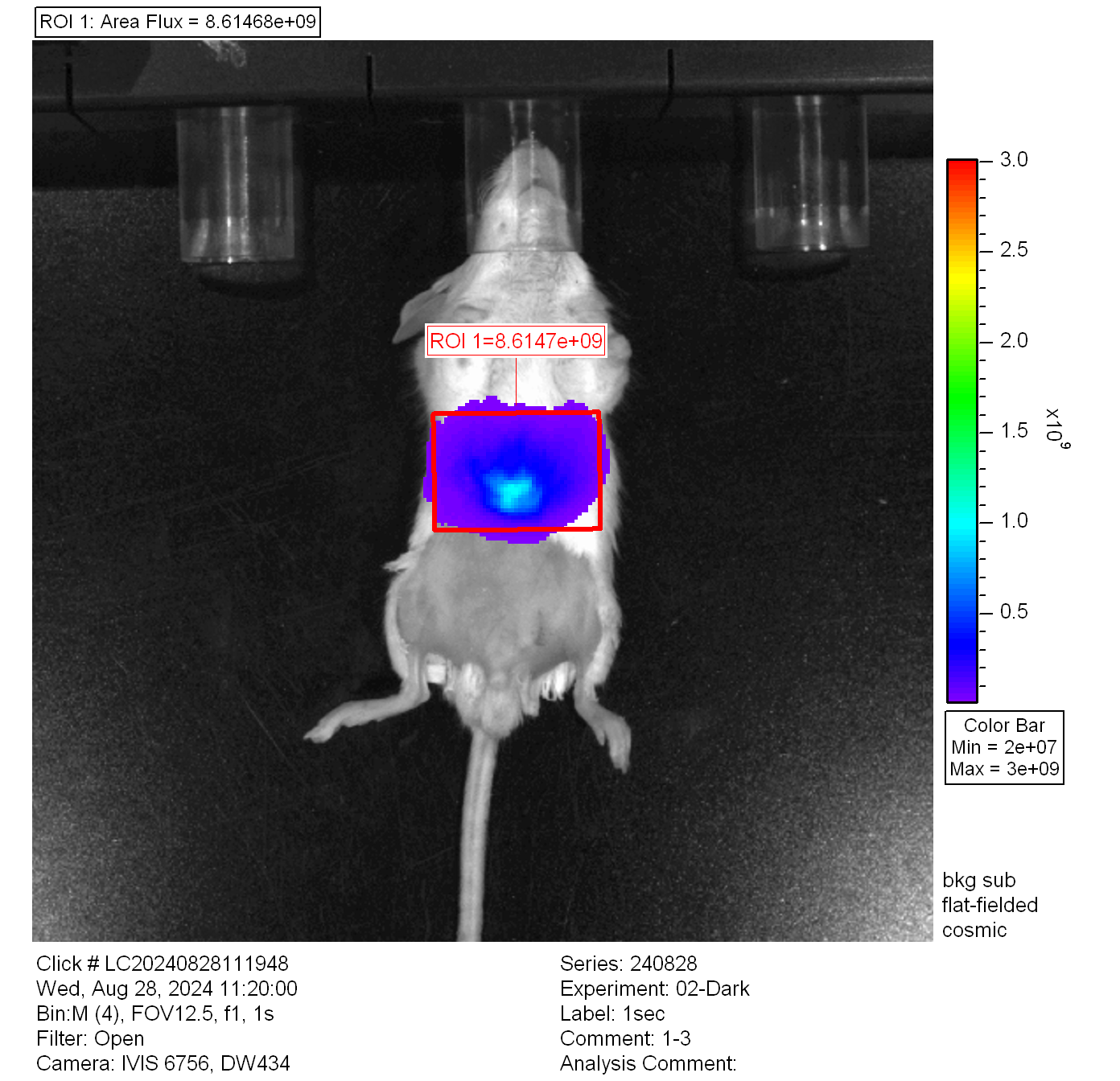

Supplement: Supplementary file 10 — Source Data [file 41467_2025_61322_MOESM10_ESM.zip › Source Data - Figure 6b/02_mRNA-RELISR(Dark)_composite.PNG]

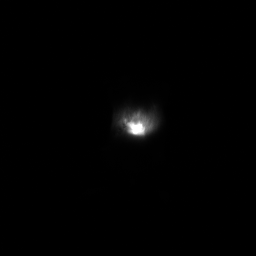

Supplement: Supplementary file 10 — Source Data [file 41467_2025_61322_MOESM10_ESM.zip › Source Data - Figure 6b/02_mRNA-RELISR(Dark)_Luminescent.TIF]

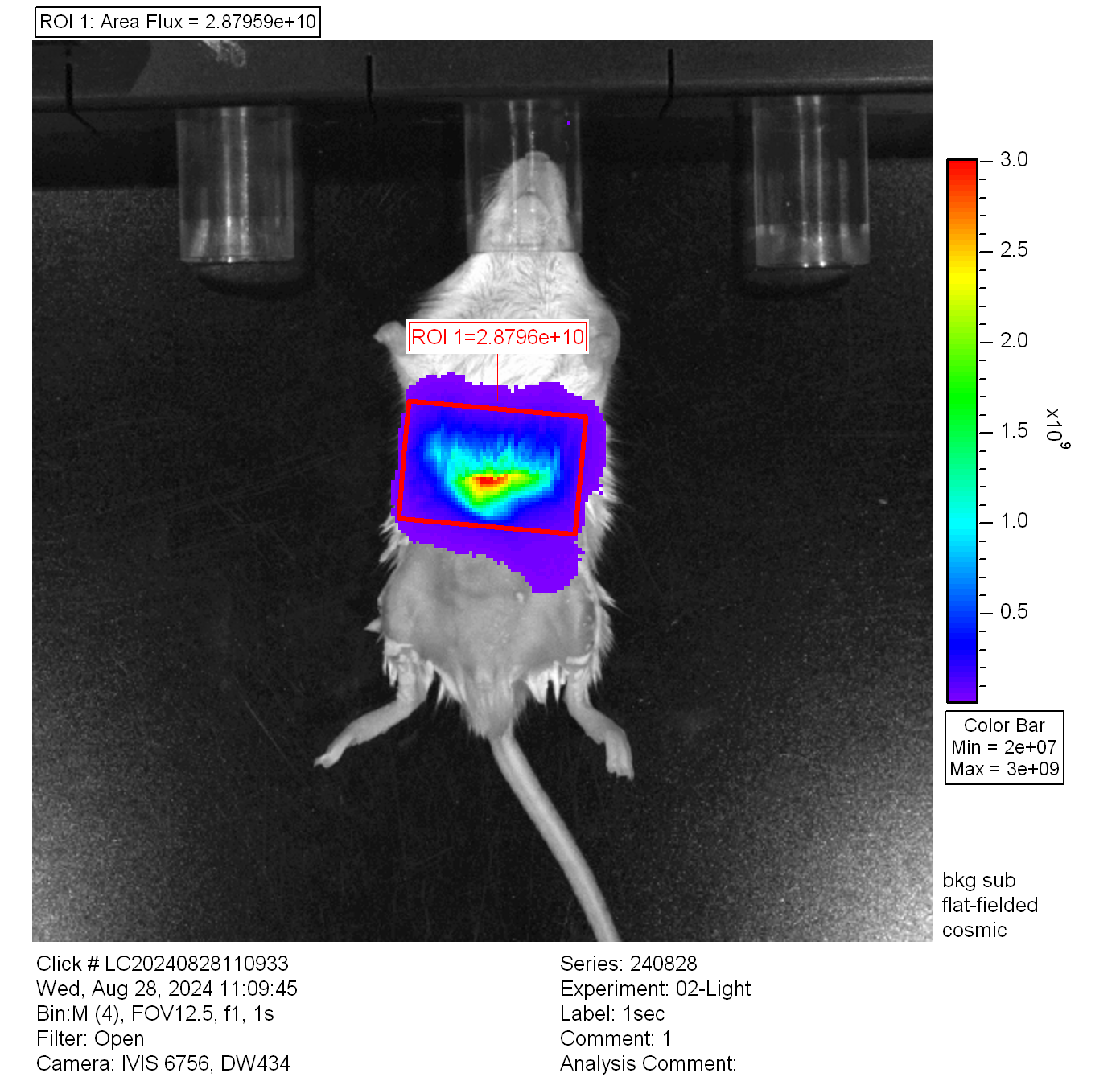

Supplement: Supplementary file 10 — Source Data [file 41467_2025_61322_MOESM10_ESM.zip › Source Data - Figure 6b/03_mRNA-RELISR(Light)_composite.PNG]

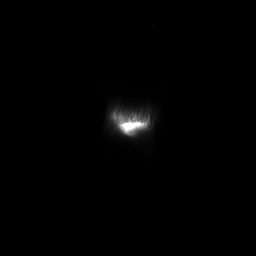

Supplement: Supplementary file 10 — Source Data [file 41467_2025_61322_MOESM10_ESM.zip › Source Data - Figure 6b/03_mRNA-RELISR(Light)_Luminescent.TIF]
